# Spirulina Supplements Improved the Nutritional Status of Undernourished Children Quickly and Significantly: Experience from Kisantu, the Democratic Republic of the Congo

**DOI:** 10.1155/2016/1296414

**Published:** 2016-09-29

**Authors:** Féfé Khuabi Matondo, Kikuni Takaisi, Adolphine Bedi Nkuadiolandu, Aimé Kazadi Lukusa, Michel Ntetani Aloni

**Affiliations:** ^1^Faculty of Medicine, Kongo University, Kisantu, Democratic Republic of the Congo; ^2^Faculty of Pharmaceutical Sciences, University of Kinshasa, Kinshasa, Democratic Republic of the Congo; ^3^Division of Nutrition, Gastroenterology and Neurology, Department of Paediatrics, University Hospital of Kinshasa, Faculty of Medicine, University of Kinshasa, Kinshasa, Democratic Republic of the Congo; ^4^Division of Hemato-Oncology and Nephrology, Department of Paediatrics, University Hospital of Kinshasa, Faculty of Medicine, University of Kinshasa, Kinshasa, Democratic Republic of the Congo

## Abstract

*Aim*. Despite high levels of malnutrition, there is still very little information on the nutritional benefits of* Spirulina*, a natural alga that provides essential amino acids, rare essential lipids, and numerous minerals and vitamins, to undernourished children in the world.* Methods*. We carried out a prospective study of 50 children aged between six and 60 months. The intervention group consisted of 16 children who received 10 g of* Spirulina* daily, as well as the local diet administered by the nutritional centre, and the control group of 34 children who just received the local diet. Both groups of children were assessed on day zero, day 15, and day 30.* Results*. After treatment, the weight-for-age *Z* scores and weight-for-height *Z* scores increased significantly in the intervention group. At day 15, there was a statistically significant difference between the mean corpuscular volume, total proteins, and albumin (*p* < 0.05) in both groups, in favour of the intervention group, and at day 30, this difference extended to all of the studied parameters (*p* < 0.05).* Conclusion*. This study found that the nutritional status of undernourished children who received* Spirulina* supplements as well as the local diet administered by the nutritional centre improved quickly and significantly.

## 1. Introduction

Malnutrition remains a major cause of morbidity and is the most common worldwide cause of death in children who are less than five years of age [1, 2]. The disease accounts for 60% of all childhood mortality in developing countries. It is estimated that 146 million children under the age of five are underweight in developing countries, compared to normal anthropometric parameters, and 3.5 million children a year die from malnutrition [1–3].

Sub-Saharan Africa remains the region most affected by malnutrition, with an estimated mortality rate of one million per year and the majority of these deaths taking place in central, west, and eastern Africa [2].

In the Democratic Republic of the Congo (DRC), 80% of the population lives in extreme poverty and more than 70% are estimated to suffer from malnutrition, with a very high rate of mortality in the paediatric population. Furthermore, 45% of children under the age of five suffer from stunted growth and 20% of children die before their fifth birthday.

Treatment regimes are based on inpatient and outpatient programmes that offer intensive medical and nutritional protocols using various types of food and studies have showed that these result in good outcomes. Both infant formulas and solid foods should contain high-quality proteins, minerals, and vitamins.

The* Spirulina* family of* Arthrospira platensis* grows naturally as an alga in the alkaline waters of some lakes in the tropics [4, 5] and has traditionally been consumed worldwide [6]. Most reports about the phytochemical screening of* Spirulina* have shown various nutritional properties, mainly essential amino acids; rare essential lipids such as gamma linolenic acid; mineral salts such as calcium, phosphorus, magnesium, zinc, copper, iron, chromium, manganese, sodium, potassium, and selenium; and vitamins such as beta carotene, a precursor of vitamin A, vitamins B1, B2, B6, B12, C, and E and enzymes [6–8].

Because* Spirulina* is digestible and contains a high level of nucleic acids, it is one of the best ingredients for high-quality food supplements [6, 7].

Despite the high prevalence of malnutrition in the paediatric population of the Democratic Republic of the Congo (DRC), there is very little information on the clinical, laboratory features and outcomes of malnourished children receiving* Spirulina* dietary supplements. It is necessary to study the main characteristics of malnourished children receiving* Spirulina* in the DRC as a starting point for future research. The findings could mean that the current general public health and preventive policies in place to tackle malnutrition are not appropriate.

We hypothesised that supplementing the local diet with* Spirulina* could improve the nutritional status of children who were underweight. A prospective study was conducted in children who were less than five years of age in the semirural area of Kisantu, which is in the province of Bas-Congo and located in the western area of the DRC. Our findings were compared to the results of previous studies reported in the literature.

## 2. Methods

### 2.1. Study Setting and Design

A case-control study was conducted from March 2008 to August 2008 in the Centre de Nutrition Thérapeutique of Kisantu, which is situated in the western part of the DRC and is the only nutritional centre operating in the rural health area of Kisantu. The centre has 80 beds and receives all malnourished children from the rural health area of Kisantu.

We consecutively recruited undernourished children between the age of six months and 60 months hospitalised during the study period, according to the 2006 World Health Organization (WHO) Growth Standards and *Z* score criteria [9].

A complete physical examination was carried out on each child by a physician. We excluded children with positive* Plasmodium falciparum* in thick and thin smears of peripheral blood, those who had positive symptoms and signs of severe malaria, those who tested positive for HIV, children with geohelminths, or those who were dehydrated.

For each case, at least one control attending the same centre matched for age and place of residence was recruited into the study. Controls (nonintervention group) were undernourished children who had not received Spirulina.

### 2.2. Data Collection Procedure and Analysis

We drew a 5 mL venous blood sample from each study participant into an EDTA tube, used to determine haematological parameters. Samples of urine and faeces were also collected from each child. These tests were performed in the clinical laboratory of the Saint Luc Hospital, a secondary care facility located in the city of Kisantu. According to the WHO criteria for the paediatric population, anaemia was defined as a haemoglobin concentration of less than 11 g/dL [10].

### 2.3. Study Protocol

All the undernourished children were submitted to a full staple diet composed of Nutrifil, a nutritionally complete supplementary food containing 150 g protein, 110 g fat, and 650 g carbohydrate (Nutrifil Ltd., Dublin, Ireland) or a sweet mix of carrots, rice, milk, and therapeutic proteins, a recipe produced by the centre. The meals were divided into four daily doses and given at 7 am, 11 am, 1 pm, and 5 pm.

The* Spirulina* was packaged in plastic bags, each containing 10 g of* Spirulina* dry granulate (Ami Kivu Laboratory, North Kivu, Democratic Republic of the Congo).

The study population comprised two groups. The intervention group consisted of children who received the local diet, plus 10 g of Spirulina a day, from the Centre de Nutrition Thérapeutique. The* Spirulina* was divided into two daily doses, one teaspoon in the morning and one teaspoon in the evening, and cooked with the basic food mixture. The two groups were assessed on the first day, which was day zero, day 15, and day 30. The following information was recorded and analysed: height and weight, the presence of oedema, laboratory features including haemoglobin, haematocrit, red blood cells count, and albumin.

The nutritional status of the study population was assessed using the *Z* scores of the anthropometric indices: weight-for-height *Z* score (WHZ), height-for-age *Z* score (HAZ), and weight-for-age *Z* score (WAZ). These indicators were calculated according to the references of the National Centre for Health Statistics [11].

### 2.4. Ethical Considerations

All of the participants were minors and their legal guardians provided written consent for them to take part in the study. Pending the installation of an ethical committee in the Province of Bas-Congo and at the Kisangani hospitals, the consent procedure and the study were reviewed and approved by the Provincial Health Officer, the highest ranked medical authority in the province, and the Director's Board of Centre de Nutrition Thérapeutique of Kisantu in line with the principles of the Declaration of Helsinki. The aim and the study procedures were explained to the legal guardians and they were informed that they could withdraw anytime without further obligation.

### 2.5. Data Management

The data was analysed using SPSS for Windows, version 12.0 (SPSS Inc., Chicago, Illinois, USA). The frequency of the clinical and laboratory findings was expressed as percentages and the data were expressed as means ± standard deviations (SD) when the distribution was normal and medians with ranges when the distribution was not normal. The Student's *t*-test was used to compare means differentials. A *p* value < 0.05 was considered significant.

## 3. Results

We recruited 50 undernourished children during the three-month study period. The median age of this group was 41.2 months (range; 6 months–60 months). The 16 children in the intervention group received* Spirulina* and the local diet and the 34 children in the control group only received the local diet. Among these children, 44 (88%) of undernourished patients were more than 24 months of age.

We analysed the nutritional status of the selected population using *Z* scores. The WAZ score showed that acute malnutrition affected 78% of the children and the HAZ showed that it was 84%. However, according to the WAZ only 30% were considered to have acute malnutrition. Oedema was present in 32 (64%) of the children.

Table 1 shows the laboratory findings of the 50 undernourished children at the start of the study. All of the undernourished children had anaemia, including 10 (20%) cases of severe anaemia and 40 (80%) cases of moderate anaemia.

The influence that* Spirulina* had on improving the nutritional status of the intervention group was analysed after 30 days of treatment. There was no significant difference for all indicators of malnutrition in the control group. In contrast, the WAZ and the WHZ increased significantly in the intervention group after the period of treatment (Table 2).

Table 3 shows the differential (delta) of the weight and biological parameters in the study population. The data in both groups showed that, at day 15, the differential was not statistically significant for weight, haemoglobin, and haematocrit levels (*p* > 0.05) between the two groups. However, the differential was statistically significant for mean corpuscular volume total proteins and albumin (*p* < 0.05) between the two groups. At day 30, the differential was statistically significant for all the study parameters (*p* < 0.05).

## 4. Discussion

*Spirulina* has been discussed for a couple of decades as a dietary source of micro- and macronutrients. Previous reports concerning the phytochemical screening of* Spirulina* extracts have revealed the presence of exceptional nutritional properties [6–8, 12, 13]. Our study was the first to look at the outcome of its use in Central Africa.

In this series, the greater proportion of undernourished patients were more than 24 months of age. This finding was similar to previous reports from other developing countries [14, 15]. Malnutrition usually affects children of this age as it is known that children are often weaned off breast milk between 12 and 24 months of age and is a major determinant of malnutrition in the paediatric population living in developing countries [16–19].

In this study, 78% of the undernourished children demonstrated failure to thrive and 84% had growth retardation. According to Demographic Health Survey 2007, the estimated prevalence of malnutrition among children of less than five years of age in the DRC was 34% in 1995, 31% in 2001, and 46% in 2007 [20].

*Spirulina* is rich in minerals, proteins, carotenoids, and vitamins [4]. In this pilot study, the administration of* Spirulina* at a dose of 10 g per day seemed to significantly and quickly improve the nutritional status of undernourished children in the intervention group when compared to the control group. Indeed, the rate of global acute malnutrition decreased from 30% before the* Spirulina* supplements to 20% at day 30. At the same time, the prevalence of oedema decreased from 64% to 4%. These results were in accordance with those reported by previous African studies on the beneficial effect of* Spirulina* in malnutrition [11, 12, 21–24].

In this report, the majority of children in the intervention group showed a significant increase of haemoglobin and haematocrit levels compared to control group. The prevalence of severe anaemia decreased from 20% to 6% at day 30. The laboratory features found in this cohort confirmed and extended the findings of several other studies [4, 12, 21]. Indeed, this situation may be due to the presence of vitamins, micronutrients, and the iron content of Spirulina [25], which helps to correct anaemia [23–26].

## 5. Conclusion

This study provided data about the use of* Spirulina* to tackle malnutrition in malnourished children in Central Africa, notably the Democratic Republic of the Congo. In this pilot study, the administration of* Spirulina* at a dose of 10 g per day seemed to significantly and quickly improve the nutritional status of undernourished children.

## Figures and Tables

**Table 1:** Characteristics of the study population.

Variables ± SD	Intervention group	Control group
	*n* = 16	*n* = 34
Weight (Kg)	9.2 (2.8)	11 (2.8)
Haemoglobin (g/dL)	7.8 (2.1)	9.6 (1.4)
Haematocrit (%)	24.0 (6.9)	29.0 (4.7)
Mean corpuscular volume (fL)	81.8 (15.4)	91.4 (10.3)
Total proteins (g/L)	60.2 (17.2)	61.1 (13.4)
Albumin (g/L)	31.9 (9.9)	38.8 (10.6)

**Table 2:** Anthropometrics indicators of malnutrition in the study population.

Variables	Day 0	Day 30	*p*
*Intervention group *			
WAZ	−2.72	−2.66	0.83
WHZ	−1.07	−1.04	0.9
*Control group*			
WAZ	−3.03	−2.18	0.04
WHZ	−1.44	−0.51	0.03

WAZ: weight-for-age *Z* score. WHZ: weight-for-height *Z* score.

**Table 3:** Differential (delta) of weight and biological parameters in the study population.

Variables	Intervention group *n* = 16	Control group *n* = 34	*p*
*Weight* (kg)			
Day 15	0.38 (0.93)	0.27 (0.63)	0.13
Day 30	1.17 (0.99)	0.05 (0.70)	0.001
*Haemoglobin* (g/dL)			
Day 15	0.89 (1.70)	0.21 (1.44)	0.15
Day 30	1.75 (1.4)	0.26 (1.42)	0.001
*Haematocrit* (%)			
Day 15	2.69 (5.33)	0.44 (4.72)	0.14
Day 30	4.25 (4.22)	0.12 (4.26)	0.002
*MCV* ^*∗*^ (fL)			
Day 15	7.85 (15.49)	1.4 (11.05)	0.02
Day 30	—	—	
*Total proteins* (g/L)			
Day 15	14.76 (7.69)	0.91 (7.64)	0.000
Day 30	—	—	
*Albumin* (g/L)			
Day 15	7.92 (9.15)	1.79 (9.84)	0.04
Day 30	—	—	

^*∗*^MCV: mean corpuscular volume.

## Abbreviations

DRC: Democratic Republic of the Congo
HAZ: Height-for-age *Z* score
WAZ: Weight-for-age *Z* score
WHZ: Weight-for-height *Z* score.
